# Levels of Reading Comprehension in Higher Education: Systematic Review and Meta-Analysis

**DOI:** 10.3389/fpsyg.2021.712901

**Published:** 2021-08-04

**Authors:** Cristina de-la-Peña, María Jesús Luque-Rojas

**Affiliations:** ^1^Departamento de Métodos de Investigación y Diagnóstico en Educación, Universidad Internacional de la Rioja, Logroño, Spain; ^2^Department of Theory and History of Education and Research Methods and Diagnosis in Education, University of Malaga, Málaga, Spain

**Keywords:** reading comprehension, higher education, university students, systematic review, meta-analysis

## Abstract

Higher education aims for university students to produce knowledge from the critical reflection of scientific texts. Therefore, it is necessary to develop a deep mental representation of written information. The objective of this research was to determine through a systematic review and meta-analysis the proportion of university students who have an optimal performance at each level of reading comprehension. Systematic review of empirical studies has been limited from 2010 to March 2021 using the Web of Science, Scopus, Medline, and PsycINFO databases. Two reviewers performed data extraction independently. A random-effects model of proportions was used for the meta-analysis and heterogeneity was assessed with *I*^2^. To analyze the influence of moderating variables, meta-regression was used and two ways were used to study publication bias. Seven articles were identified with a total sample of the seven of 1,044. The proportion of students at the literal level was 56% (95% CI = 39–72%, *I*^2^ = 96.3%), inferential level 33% (95% CI = 19–46%, *I*^2^ = 95.2%), critical level 22% (95% CI = 9–35%, *I*^2^ = 99.04%), and organizational level 22% (95% CI = 6–37%, *I*^2^ = 99.67%). Comparing reading comprehension levels, there is a significant higher proportion of university students who have an optimal level of literal compared to the rest of the reading comprehension levels. The results have to be interpreted with caution but are a guide for future research.

## Introduction

Reading comprehension allows the integration of knowledge that facilitates training processes and successful coping with academic and personal situations. In higher education, this reading comprehension has to provide students with autonomy to self-direct their academic-professional learning and provide critical thinking in favor of community service (UNESCO, 2009). However, research in recent years (Bharuthram, 2012; Afflerbach et al., 2015) indicates that a part of university students are not prepared to successfully deal with academic texts or they have reading difficulties (Smagorinsky, 2001; Cox et al., 2014), which may limit academic training focused on written texts. This work aims to review the level of reading comprehension provided by studies carried out in different countries, considering the heterogeneity of existing educational models.

The level of reading comprehension refers to the type of mental representation that is made of the written text. The reader builds a mental model in which he can integrate explicit and implicit data from the text, experiences, and previous knowledge (Kucer, 2016; van den Broek et al., 2016). Within the framework of the construction-integration model (Kintsch and van Dijk, 1978; Kintsch, 1998), the most accepted model of reading comprehension, processing levels are differentiated, specifically: A superficial level that identifies or memorizes data forming the basis of the text and a deep level in which the text situation model is elaborated integrating previous experiences and knowledge. At these levels of processing, the cognitive strategies used, are different according to the domain-learning model (Alexander, 2004) from basic coding to a transformation of the text. In the scientific literature, there are investigations (Yussof et al., 2013; Ulum, 2016) that also identify levels of reading comprehension ranging from a literal level of identification of ideas to an inferential and critical level that require the elaboration of inferences and the data transformation.

Studies focused on higher education (Barletta et al., 2005; Yáñez Botello, 2013) show that university students are at a literal or basic level of understanding, they often have difficulties in making inferences and recognizing the macrostructure of the written text, so they would not develop a model of a situation of the text. These scientific results are in the same direction as the research on reading comprehension in the mother tongue in the university population. Bharuthram (2012) indicates that university students do not access or develop effective strategies for reading comprehension, such as the capacity for abstraction and synthesis-analysis. Later, Livingston et al. (2015) find that first-year education students present limited reading strategies and difficulties in understanding written texts. Ntereke and Ramoroka (2017) found that only 12.4% of students perform well in a reading comprehension task, 34.3% presenting a low level of execution in the task.

Factors related to the level of understanding of written information are the mode of presentation of the text (printed vs. digital), the type of metacognitive strategies used (planning, making inferences, inhibition, monitoring, etc.), the type of text and difficulties (novel vs. a science passage), the mode of writing (text vs. multimodal), the type of reading comprehension task, and the diversity of the student. For example, several studies (Tuncer and Bahadir, 2014; Trakhman et al., 2019; Kazazoglu, 2020) indicate that reading is more efficient with better performance in reading comprehension tests in printed texts compared to the same text in digital and according to Spencer (2006) college students prefer to read in print vs. digital texts. In reading the written text, metacognitive strategies are involved (Amril et al., 2019) but studies (Channa et al., 2018) seem to indicate that students do not use them for reading comprehension, specifically; Korotaeva (2012) finds that only 7% of students use them. Concerning the type of text and difficulties, for Wolfe and Woodwyk (2010), expository texts benefit more from the construction of a situational model of the text than narrative texts, although Feng (2011) finds that expository texts are more difficult to read than narrative texts. Regarding the modality of the text, Mayer (2009) and Guo et al. (2020) indicate that multimodal texts that incorporate images into the text positively improve reading comprehension. In a study of Kobayashi (2002) using open questions, close, and multiple-choice shows that the type and format of the reading comprehension assessment test significantly influence student performance and that more structured tests help to better differentiate the good ones and the poor ones in reading comprehension. Finally, about student diversity, studies link reading comprehension with the interest and intrinsic motivation of university students (Cartwright et al., 2019; Dewi et al., 2020), with gender (Saracaloglu and Karasakaloglu, 2011), finding that women present a better level of reading comprehension than men and with knowledge related to reading (Perfetti et al., 1987). In this research, it was controlled that all were printed and unimodal texts, that is, only text. This is essential because the cognitive processes involved in reading comprehension can vary with these factors (Butcher and Kintsch, 2003; Xu et al., 2020).

## The Present Study

Regardless of the educational context, in any university discipline, preparing essays or developing arguments are formative tasks that require a deep level of reading comprehension (inferences and transformation of information) that allows the elaboration of a situation model, and not having this level can lead to limited formative learning. Therefore, the objective of this research was to know the state of reading comprehension levels in higher education; specifically, the proportion of university students who perform optimally at each level of reading comprehension. It is important to note that there is not much information about the different levels in university students and that it is the only meta-analytic review that explores different levels of reading comprehension in this educational stage. This is a relevant issue because the university system requires that students produce knowledge from the critical reflection of scientific texts, preparing them for innovation, employability, and coexistence in society.

## Materials and Methods

### Eligibility Criteria: Inclusion and Exclusion

Empirical studies written in Spanish or English are selected that analyze the reading comprehension level in university students.

The exclusion criteria are as follows: (a) book chapters or review books or publications; (b) articles in other languages; (c) studies of lower educational levels; (d) articles that do not identify the age of the sample; (e) second language studies; (f) students with learning difficulties or other disorders; (g) publications that do not indicate the level of reading comprehension; (h) studies that relate reading competence with other variables but do not report reading comprehension levels; (i) pre-post program application work; (j) studies with experimental and control groups; (k) articles comparing pre-university stages or adults; (l) publications that use multi-texts; (m) studies that use some type of technology (computer, hypertext, web, psychophysiological, online questionnaire, etc.); and (n) studies unrelated to the subject of interest.

Only those publications that meet the following criteria are included as: (a) be empirical research (article, thesis, final degree/master’s degree, or conference proceedings book); (b) university stage; (c) include data or some measure on the level of reading comprehension that allows calculating the effect size; (d) written in English or Spanish; (e) reading comprehension in the first language or mother tongue; and (f) the temporary period from January 2010 to March 2021.

### Search Strategies

A three-step procedure is used to select the studies included in the meta-analysis. In the first step, a review of research and empirical articles in English and Spanish from January 2010 to March 2021. The search is carried out in online databases of languages in Spanish and English, such as Web of Science (WoS), Scopus, Medline, and PsycINFO, to review empirical productions that analyze the level of reading comprehension in university students. In the second step, the following terms (titles, abstracts, keywords, and full text) are used to select the articles: Reading comprehension and higher education, university students, in Spanish and English, combined with the Boolean operators AND and OR. In the last step, secondary sources, such as the Google search engine, Theseus, and references in publications, are explored.

The search reports 4,294 publications (articles, theses, and conference proceedings books) in the databases and eight records of secondary references, specifically, 1989 from WoS, 2001 from Scopus, 42 from Medline, and 262 of PsycINFO. Of the total (4,294), 1,568 are eliminated due to duplications, leaving 2,734 valid records. Next, titles and abstracts are reviewed and 2,659 are excluded because they do not meet the inclusion criteria. The sample of 75 publications is reduced to 40 articles, excluding 35 because the full text cannot be accessed (the authors were contacted but did not respond), the full text did not show specific statistical data, they used online questionnaires or computerized presentations of the text. Finally, seven articles in Spanish were selected for use in the meta-analysis of the reading comprehension level of university students. Data additional to those included in the articles were not requested from the selected authors.

**Figure 1:**
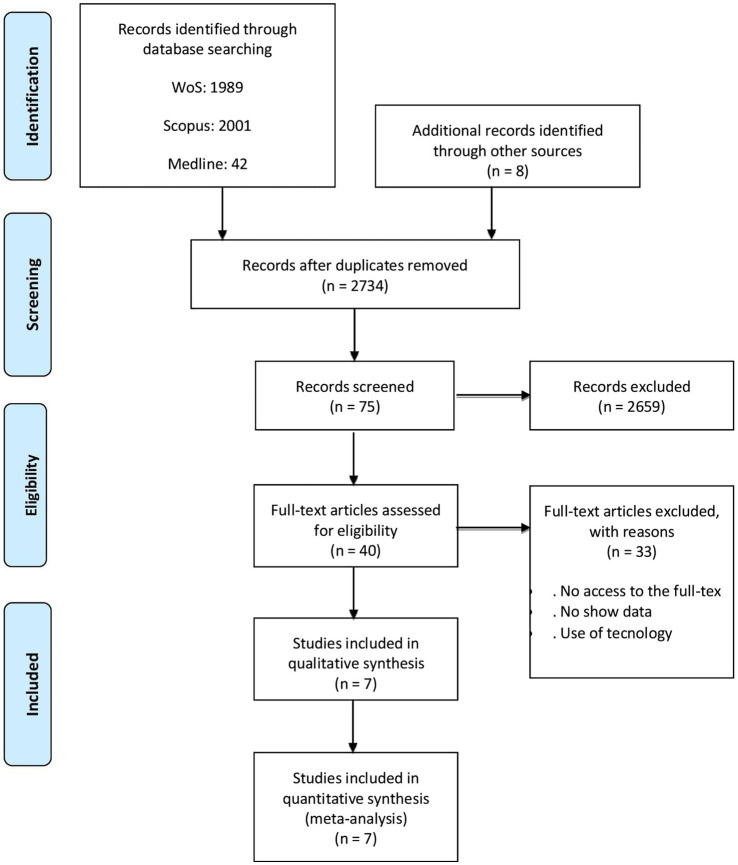
Flow diagram for the selection of articles.

The PRISMA-P guidelines (Moher et al., 2015) are followed to perform the meta-analysis and the flow chart for the selection of publications relevant to the subject is exposed (Figure 1).

### Encoding Procedure

This research complies with what is established in the manual of systematic reviews (Higgins and Green, 2008) in which clear objectives, specific search terms, and eligibility criteria for previously defined works are established. Two independent coders, reaching a 100% agreement, carry out the study search process. Subsequently, the research is codified, for this, a coding protocol is used as a guide to help resolve the ambiguities between the coders; the proposals are reflected and discussed and discrepancies are resolved, reaching a degree of agreement between the two coders of 97%.

For all studies, the reference, country, research objective, sample size, age and gender, reading comprehension test, other tests, and reading comprehension results were coded in percentages. All this information was later systematized in Table 1.

**Table 1:** Results of the empirical studies included in the meta-analysis.

S. No.	Reference	Country	Objective	Sample (*n*°/age/sex)	Comprehension Instrumentos	Other tests	Reading comprehension results
1.	Del Pino-Yépez et al., 2019	Ecuador	Assess the effectiveness of didactic strategies to strengthen the level of reading comprehension	30 Educación/unknown/unknown	*Ad hoc* text with 12SS questions		Literal: 40% Inferential: 40% Critical: 20%
2.	Guevara Benítez et al., 2014	México	Validate reading comprehension test	570 Psychology/19.9 years/72% women 28% men	Instrument to measure reading comprehension of university students (ICLAU)		Literal: 41% Inferential: 33% Critical: 47% Appreciative: 72% Organization.: 75% Prueba general: 66%
3.	Sáez Sánchez, 2018	México	Assess reading comprehension	101 Education/unknown/unknown	*Ad hoc* text with questions		Literal: 52.86% Inferential: 52.92% Critical: 53.89% Organization: 66.46% Appreciative: 44.01%
4.	Sanabria Mantilla, 2018	Bolivia	Identify the relationship between reading comprehension and academic performance	49 Psychology/18.5 years/87.8% women 12.2% men	Instrument to measure reading comprehension in university students (ICLAU)	Academic qualifications	Literal: 67.3% Inferential: 12.2% Organization: 4.1% Critical: 0% Appreciative: 0%
5.	Márquez et al., 2016	Chile	Know the level of reading comprehension	44 Kinesiology and Nutrition and Dietetics/unknown/unknown	Instrument to measure reading comprehension in university students (ICLAU)		Literal: 43.2% Inferential: 4.5% Critical: 0% Organization: 4.5%
6.	Yáñez Botello, 2013	Colombia	Characterize the cognitive processes involved in reading and their relationship with reading comprehension levels	124 Psychology/16–30 years/unknown	Arenas Reading Comprehension Assessment Questionnaire (2007)		Literal: 56.4% Inferential: 43.5% Critical: 0%
7.	Figueroa Romero et al., 2016	Perú	Determine the level of reading comprehension	126 from the 1°year University/43% men and 57% women/15–26 years	Reading comprehension test 10 fragments with 28 questions	Bibliographic datasheet	Literal: 86.7% Inferential: 45.4% Critical: 34.29%

In relation to the type of reading comprehension level, it was coded based on the levels of the scientific literature as follows: 1 = literal; 2 = inferential; 3 = critical; and 4 = organizational.

Regarding the possible moderating variables, it was coded if the investigations used a standardized reading comprehension measure (value = 1) or non-standardized (value = 0). This research considers the standardized measures of reading comprehension as the non-standardized measures created by the researchers themselves in their studies or questionnaires by other authors. By the type of evaluation test, we encode between multiple-choice (value = 0) or multiple-choices plus open question (value = 1). By type of text, we encode between argumentative (value = 1) or unknown (value = 0). By the type of career, we encode social sciences (value = 1) or other careers (health sciences; value = 0). Moreover, by the type of publication, we encode between article (value = 1) or doctoral thesis (value = 0).

### Effect Size and Statistical Analysis

This descriptive study with a sample *k* = 7 and a population of 1,044 university students used a continuous variable and the proportions were used as the effect size to analyze the proportion of students who had an optimal performance at each level of reading comprehension. As for the percentages of each level of reading comprehension of the sample, they were transformed into absolute frequencies. A random-effects model (Borenstein et al., 2009) was used as the effect size. These random-effects models have a greater capacity to generalize the conclusions and allow estimating the effects of different sources of variation (moderating variables). The DerSimonian and Laird method (Egger et al., 2001) was used, calculating raw proportion and for each proportion its standard error, value of *p* and 95% confidence interval (CI).

To examine sampling variability, Cochran’s *Q* test (to test the null hypothesis of homogeneity between studies) and *I*^2^ (proportion of variability) were used. According to Higgins et al. (2003), if *I*^2^ reaches 25%, it is considered low, if it reaches 50% and if it exceeds 75% it is considered high. A meta-regression analysis was used to investigate the effect of the moderator variables (type of measure, type of evaluation test, type of text, type of career, and type of publication) in each level of reading comprehension of the sample studies. For each moderating variable, all the necessary statistics were calculated (estimate, standard error, CI, *Q*, and *I*^2^).

To compare the effect sizes of each level (literal, inferential, critical, and organizational) of reading comprehension, the chi-square test for the proportion recommended by Campbell (2007) was used.

Finally, to analyze publication bias, this study uses two ways: Rosenthal’s fail-safe number and regression test. Rosenthal’s fail-safe number shows the number of missing studies with null effects that would make the previous correlations insignificant (Borenstein et al., 2009). When the values are large there is no bias. In the regression test, when the regression is not significant, there is no bias.

The software used to classify and encode data and produce descriptive statistics was with Microsoft Excel and the Jamovi version 1.6 free software was used to perform the meta-analysis.

## Results

The results of the meta-analysis are presented in three parts: the general descriptive analysis of the included studies; the meta-analytic analysis with the effect size, heterogeneity, moderating variables, and comparison of effect sizes; and the study of publication bias.

### Overview of Included Studies

The search carried out of the scientific literature related to the subject published from 2010 to March 2021 generated a small number of publications, because it was limited to the higher education stage and required clear statistical data on reading comprehension.

Table 1 presents all the publications reviewed in this meta-analysis with a total of students evaluated in the reviewed works that amounts to 1,044, with the smallest sample size of 30 (Del Pino-Yépez et al., 2019) and the largest with 570 (Guevara Benítez et al., 2014). Regarding gender, 72% women and 28% men were included. Most of the sample comes from university degrees in social sciences, such as psychology and education (71.42%) followed by health sciences (14.28%) engineering and a publication (14.28%) that does not indicate origin. These publications selected according to the inclusion criteria for the meta-analysis come from more countries with a variety of educational systems, but all from South America. Specifically, the countries that have more studies are Mexico (28.57%) and Colombia, Chile, Bolivia, Peru, and Ecuador with 14.28% each, respectively. The years in which they were published are 2.57% in 2018 and 2016 and 14.28% in 2019, 2014, and 2013.

A total of 57% of the studies analyze four levels of reading comprehension (literal, inferential, critical, and organizational) and 43% investigate three levels of reading comprehension (literal, inferential, and critical). Based on the moderating variables, 57% of the studies use standardized reading comprehension measures and 43% non-standardized measures. According to the evaluation test used, 29% use multiple-choice questions and 71% combine multiple-choice questions plus open questions. 43% use an argumentative text and 57% other types of texts (not indicated in studies). By type of career, 71% are students of social sciences and 29% of other different careers, such as engineering or health sciences. In addition, 71% are articles and 29% with research works (thesis and degree works).

Table 2 shows the reading comprehension assessment instruments used by the authors of the empirical research integrated into the meta-analysis.

**Table 2:** Reading comprehension assessment tests used in higher education.

Studies	Evaluation tests	Description	Validation/Baremation
Del Pino-Yépez et al., 2019	*Ad hoc* text with 12 questions	Text “Narcissism” with 12 questions: 4 literal, 4 inferential, and 4 critical. 40 min	Validation: no Reliability: no Baremation: no
Guevara Benítez et al., 2014; Márquez et al., 2016; Sanabria Mantilla, 2018	Instrument to measure reading comprehension in university students (ICLAU)	965-word text on “Evolution and its history.” Then 7 questions are answered as: 2 literal, 2 inferential, 1 organizational, 1 critical, and 1 appreciative. 1 h	Inter-judge validation Reliability: no Baremation: no
Sáez Sánchez, 2018	*Ad hoc* text with questions	596-word text on “Ausubel’s theory.” Then literal, inferential, organizational, appreciative, and critical level questions	Validation: no Reliability: no Baremation: no
Yáñez Botello, 2013	Arenas Reading Comprehension Assessment Questionnaire (2007)	Texts 4: 2 literary and 2 scientific with 32 questions each and four answer options	Inter-judge validation Reliability: no Baremation: no
Figueroa Romero et al., 2016	Reading comprehension test by Violeta Tapia Mendieta and Maritza Silva Alejos	35 min	Validation: empirical validity: 0.58 Reliability: test-retest: 0.53 Baremation: yes

### Meta-Analytic Analysis of the Level of Reading Comprehension

**Figure 2:**
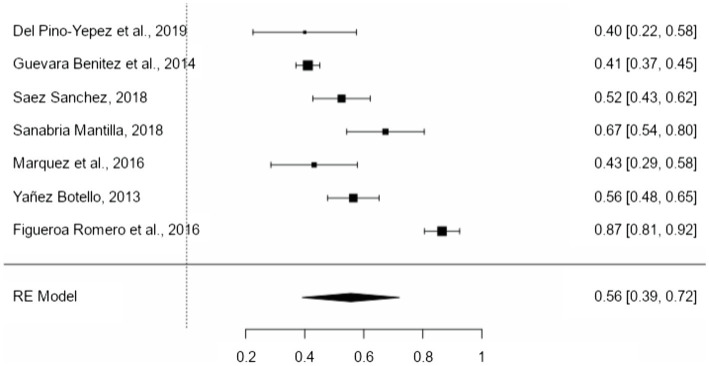
Forest plot of literal level.

The literal level presents a mean proportion effect size of 56% (95% CI = 39–72%; Figure 2). The variability between the different samples of the literal level of reading comprehension was significant (*Q* = 162.066, *p* < 0.001; *I*^2^ = 96.3%). No moderating variable used in this research had a significant contribution to heterogeneity: type of measurement (*p* = 0.520), type of test (*p* = 0.114), type of text (*p* = 0.520), type of career (*p* = 0.235), and type of publication (*p* = 0.585). The high variability is explained by other factors not considered in this work, such as the characteristics of the students (cognitive abilities) or other issues.

**Figure 3:**
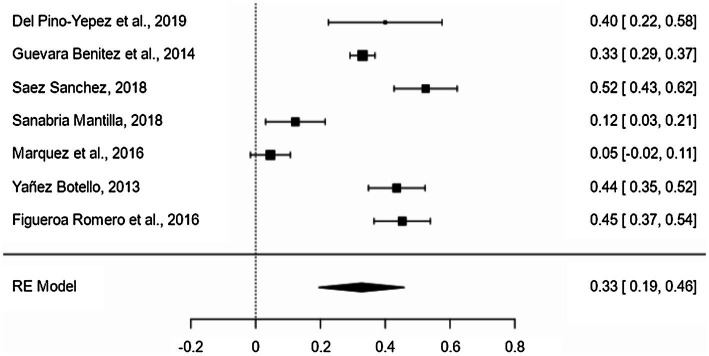
Forest plot of inferential level.

The inferential level presents a mean proportion effect size of 33% (95% CI = 19–46%; Figure 3). The variability between the different samples of the inferential level of reading comprehension was significant (*Q* = 125.123, *p* < 0.001; *I*^2^ = 95.2%). The type of measure (*p* = 0.011) and the type of text (*p* = 0.011) had a significant contribution to heterogeneity. The rest of the variables had no significance: type of test (*p* = 0.214), type of career (*p* = 0.449), and type of publication (*p* = 0.218). According to the type of measure, the proportion of students who have an optimal level in inferential administering a standardized test is 28.7% less than when a non-standardized test is administered. The type of measure reduces variability by 2.57% and explains the differences between the results of the studies at the inferential level. According to the type of text, the proportion of students who have an optimal level in inferential using an argumentative text is 28.7% less than when using another type of text. The type of text reduces the variability by 2.57% and explains the differences between the results of the studies at the inferential level.

**Figure 4:**
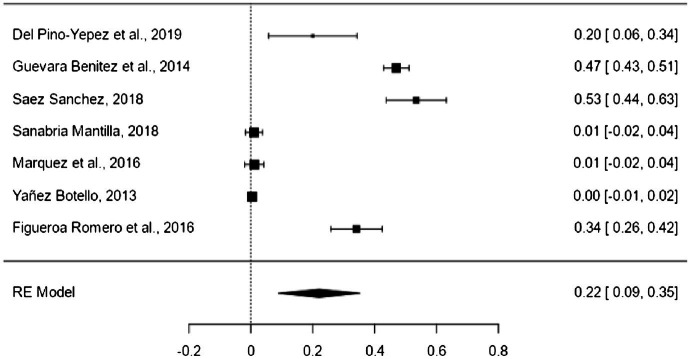
Forest plot of critical level.

The critical level has a mean effect size of the proportion of 22% (95% CI = 9–35%; Figure 4). The variability between the different samples of the critical level of reading comprehension was significant (*Q* = 627.044, *p* < 0.001; *I*^2^ = 99.04%). No moderating variable used in this research had a significant contribution to heterogeneity: type of measurement (*p* = 0.575), type of test (*p* = 0.691), type of text (*p* = 0.575), type of career (*p* = 0.699), and type of publication (*p* = 0.293). The high variability is explained by other factors not considered in this work, such as the characteristics of the students (cognitive abilities).

**Figure 5:**
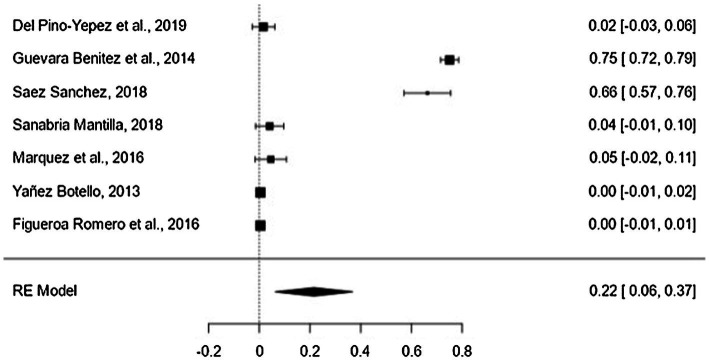
Forest plot of organizational level.

The organizational level presents a mean effect size of the proportion of 22% (95% CI = 6–37%; Figure 5). The variability between the different samples of the organizational level of reading comprehension was significant (*Q* = 1799.366, *p* < 0.001; *I*^2^ = 99.67%). The type of test made a significant contribution to heterogeneity (*p* = 0.289). The other moderating variables were not significant in this research: type of measurement (*p* = 0.289), type of text (*p* = 0.289), type of career (*p* = 0.361), and type of publication (*p* = 0.371). Depending on the type of test, the proportion of students who have an optimal level in organizational with multiple-choices tests plus open questions is 37% higher than while using only multiple-choice tests. The type of text reduces the variability by 0.27% and explains the differences between the results of the studies at the organizational level.

Table 3 shows the difference between the estimated effect sizes and the significance. There is a larger proportion of students having an optimal level of reading comprehension at the literal level compared to the inferential, critical, and organizational level; an optimal level of reading comprehension at the inferential level vs. the critical and organizational level.

**Table 3:** Results of effect size comparison.

	*X* ^2^	Difference	CI	Value of *p*
Literal-Inferential	110.963	22.9%	18.7035–26.9796%	*p* < 0.0001
Literal-Critical	248.061	33.6%	25.5998–37.4372%	*p* < 0.0001
Literal-Organizational	264.320	34.6%	30.6246–38.4088%	*p* < 0.0001
Inferential-Critical	30.063	10.7%	6.865–14.4727%	*p* < 0.0001
Inferential-Organizational	36.364	11.7%	7.9125–15.4438%	*p* < 0.0001
Critical-Organizational	0.309	1%	−2.5251–4.5224%	*p* = 0.5782

### Analysis of Publication Bias

This research uses two ways to verify the existence of bias independently of the sample size. Table 4 shows the results and there is no publication bias at any level of reading comprehension.

**Table 4:** Publication bias results.

	Fail-safe N	Value of *p*	Regression test	Value of *p*
Literal	3115.000	<0.001	−0.571	0.568
Inferential	1145.000	<0.001	0.687	0.492
Critical	783.000	<0.001	1.862	0.063
Organizational	1350.000	<0.001	1.948	0.051

## Discussion

This research used a systematic literature search and meta-analysis to provide estimates of the number of cases of university students who have an optimal level in the different levels of reading comprehension. All the information available on the subject at the international level was analyzed using international databases in English and Spanish, but the potentially relevant publications were limited. Only seven Spanish language studies were identified internationally. In these seven studies, the optimal performance at each level of reading comprehension varied, finding heterogeneity associated with the very high estimates, which indicates that the summary estimates have to be interpreted with caution and in the context of the sample and the variables used in this meta-analysis.

In this research, the effects of the type of measure, type of test, type of text, type of career, and type of publication have been analyzed. Due to the limited information in the publications, it was not possible to assess the effect of any more moderating variables.

We found that some factors significantly influence heterogeneity according to the level of reading comprehension considered. The type of measure influenced the optimal performance of students in the inferential level of reading comprehension; specifically, the proportion of students who have an optimal level in inferential worsens if the test is standardized. Several studies (Pike, 1996; Koretz, 2002) identify differences between standardized and non-standardized measures in reading comprehension and a favor of non-standardized measures developed by the researchers (Pyle et al., 2017). The ability to generate inferences of each individual may difficult to standardize because each person differently identifies the relationship between the parts of the text and integrates it with their previous knowledge (Oakhill, 1982; Cain et al., 2004). This mental representation of the meaning of the text is necessary to create a model of the situation and a deep understanding (McNamara and Magliano, 2009; van den Broek and Espin, 2012).

The type of test was significant for the organizational level of reading comprehension. The proportion of students who have an optimal level in organizational improves if the reading comprehension assessment test is multiple-choice plus open questions. The organizational level requires the reordering of written information through analysis and synthesis processes (Guevara Benítez et al., 2014); therefore, it constitutes a production task that is better reflected in open questions than in reproduction questions as multiple choice (Dinsmore and Alexander, 2015). McNamara and Kintsch (1996) identify that open tasks require an effort to make inferences related to previous knowledge and multidisciplinary knowledge. Important is to indicate that different evaluation test formats can measure different aspects of reading comprehension (Zheng et al., 2007).

The type of text significantly influenced the inferential level of reading comprehension. The proportion of students who have an optimal level in inferential decreases with an argumentative text. The expectations created before an argumentative text made it difficult to generate inferences and, therefore, the construction of the meaning of the text. This result is in the opposite direction to the study by Diakidoy et al. (2011) who find that the refutation text, such as the argumentative one, facilitates the elaboration of inferences compared to other types of texts. It is possible that the argumentative text, given its dialogical nature of arguments and counterarguments, with a subject unknown by the students, has determined the decrease of inferences based on their scarce previous knowledge of the subject, needing help to elaborate the structure of the text read (Reznitskaya et al., 2007). It should be pointed out that in meta-analysis studies, 43% use argumentative texts. Knowing the type of the text is relevant for generating inferences, for instance, according to Baretta et al. (2009) the different types of text are processed differently in the brain generating more or fewer inferences; specifically, using the N400 component, they find that expository texts generate more inferences from the text read.

For the type of career and the type of publication, no significance was found at any level of reading comprehension in this sample. This seems to indicate that university students have the same level of performance in tasks of literal, critical inferential, and organizational understanding regardless of whether they are studying social sciences, health sciences, or engineering. Nor does the type of publication affect the state of the different levels of reading comprehension in higher education.

The remaining high heterogeneity at all levels of reading comprehension was not captured in this review, indicating that there are other factors, such as student characteristics, gender, or other issues, that are moderating and explaining the variability at the literal, inferential, critical, and organizational reading comprehension in university students.

To the comparison between the different levels of reading comprehension, the literal level has a significantly higher proportion of students with an optimal level than the inferential, critical, and organizational levels. The inferential level has a significantly higher proportion of students with an optimal level than the critical and organizational levels. This corresponds with data from other investigations (Márquez et al., 2016; Del Pino-Yépez et al., 2019) that indicate that the literal level is where university students execute with more successes, being more difficult and with less success at the inferential, organizational, and critical levels. This indicates that university students of this sample do not generate a coherent situation model that provides them with a global mental representation of the read text according to the model of Kintsch (1998), but rather they make a literal analysis of the explicit content of the read text. This level of understanding can lead to less desirable results in educational terms (Dinsmore and Alexander, 2015).

The educational implications of this meta-analysis in this sample are aimed at making universities aware of the state of reading comprehension levels possessed by university students and designing strategies (courses and workshops) to optimize it by improving the training and employability of students. Some proposals can be directed to the use of reflection tasks, integration of information, graphic organizers, evaluation, interpretation, nor the use of paraphrasing (Rahmani, 2011). Some studies (Hong-Nam and Leavell, 2011; Parr and Woloshyn, 2013) demonstrate the effectiveness of instructional courses in improving performance in reading comprehension and metacognitive strategies. In addition, it is necessary to design reading comprehension assessment tests in higher education that are balanced, validated, and reliable, allowing to have data for the different levels of reading comprehension.

## Limitations and Conclusion

This meta-analysis can be used as a starting point to report on reading comprehension levels in higher education, but the results should be interpreted with caution and in the context of the study sample and variables. Publications without sufficient data and inaccessible articles, with a sample of seven studies, may have limited the international perspective. The interest in studying reading comprehension in the mother tongue, using only unimodal texts, without the influence of technology and with English and Spanish has also limited the review. The limited amount of data in the studies has limited meta-regression.

This review is a guide to direct future research, broadening the study focus on the level of reading comprehension using digital technology, experimental designs, second languages, and investigations that relate reading comprehension with other factors (gender, cognitive abilities, etc.) that can explain the heterogeneity in the different levels of reading comprehension. The possibility of developing a comprehensive reading comprehension assessment test in higher education could also be explored.

This review contributes to the scientific literature in several ways. In the first place, this meta-analytic review is the only one that analyzes the proportion of university students who have an optimal performance in the different levels of reading comprehension. This review is made with international publications and this topic is mostly investigated in Latin America. Second, optimal performance can be improved at all levels of reading comprehension, fundamentally inferential, critical, and organizational. The literal level is significantly the level of reading comprehension with the highest proportion of optimal performance in university students. Third, the students in this sample have optimal performance at the inferential level when they are non-argumentative texts and non-standardized measures, and, in the analyzed works, there is optimal performance at the organizational level when multiple-choice questions plus open questions are used.

The current research is linked to the research project “Study of reading comprehension in higher education” of Asociación Educar para el Desarrollo Humano from Argentina.

## Data Availability Statement

The raw data supporting the conclusions of this article will be made available by the authors, without undue reservation.

## Author Contributions

Cd-l-P had the idea for the article and analyzed the data. ML-R searched the data. Cd-l-P and ML-R selected the data and contributed to the valuable comments and manuscript writing. All authors contributed to the article and approved the submitted version.

## Conflict of Interest

The authors declare that the research was conducted in the absence of any commercial or financial relationships that could be construed as a potential conflict of interest.

The handling editor declared a shared affiliation though no other collaboration with one of the authors ML-R at the time of the review.

## Publisher’s Note

All claims expressed in this article are solely those of the authors and do not necessarily represent those of their affiliated organizations, or those of the publisher, the editors and the reviewers. Any product that may be evaluated in this article, or claim that may be made by its manufacturer, is not guaranteed or endorsed by the publisher.
